# Neurodiversity in elite sport: a systematic scoping review

**DOI:** 10.1136/bmjsem-2023-001575

**Published:** 2023-06-15

**Authors:** Erin Hoare, Jonathan Reyes, Lisa Olive, Catherine Willmott, Emma Steer, Michael Berk, Kate Hall

**Affiliations:** 1 IMPACT, The Institute for Mental and Physical Health and Clinical Translation, School of Medicine, Barwon Health, Deakin University, Geelong, VIC, Australia; 2 Australian Football League, Melbourne, Victoria, Australia; 3 Turner Institute for Brain & Mental Health, School of Psychological Sciences, Monash University, Melbourne, Victoria, Australia; 4 Monash-Epworth Rehabilitation Research Centre, Epworth Hospital, Melbourne, VIC, Australia; 5 School of Psychology, Deakin University, Burwood, Victoria, Australia; 6 Orygen, National Centre of Excellence in Youth Mental Health, Parkville, Victoria, Australia; 7 Centre of Youth Mental Health, University of Melbourne, Parkville, Victoria, Australia; 8 Clinical and Educational and Developmental Psychologist, Melbourne, Victoria, Australia; 9 Florey Institute for Neuroscience and Mental Health, University of Melbourne, Parkville, Victoria, Australia; 10 Department of Psychiatry, Royal Melbourne Hospital, University of Melbourne, Parkville, Victoria, Australia

**Keywords:** Psychiatry, Athlete, Elite performance

## Abstract

The objective of this systematic scoping review is to understand the extent and scope of evidence regarding neurodiversity in elite sport. This systematic scoping review considered epidemiological studies, com

mentary and viewpoints papers, systematic review and meta-analyses, and any intervention or clinical treatment, management and practice studies in relation to neurodiversity in elite sport. Case studies and grey literature were ineligible for review. Neurodivergence included neurodevelopmental disorders such as autism spectrum disorder, attention-deficit hyperactivity disorder (ADHD) and specific learning disorders. Elite sport was defined as Olympic, Paralympic, national, international, professional and semiprofessional sport. The final 23 studies included in this review comprised 10 observational studies, 4 systematic/narrative reviews, 6 commentary/position statements and 3 qualitative studies. The literature reflected a major focus on ADHD as a risk factor for concussion and prognosis for postconcussion recovery. Further, there was a focus on the medical management of ADHD, regarding adherence to sporting antidoping regulations. One study focused on the experience of autism in athletes in elite sport settings through qualitative interviews. One study focused on anxiety disorders in elite athletes, with ADHD emerging as a major risk factor. There is a strong rationale for future research to build on the evidence for neurodiversity in elite sport to foster supportive and inclusive elite sporting environments.

**WHAT IS ALREADY KNOWN ON THIS TOPIC**

- There is community and scholarly shift towards understanding neurodiversity from a strengths-based approach, which recognises differences in cognition, social learning and other behaviours as variations in the context of human neurodevelopment.
- There is yet to be a synthesis of evidence summarising the state and scope of evidence relating to neurodiversity in elite sport.

**WHAT THIS STUDY ADDS**

- The findings of this systematic scoping review indicate that while neurodivergence is present in elite sport, there is very limited research in this space to date.

**HOW THIS STUDY MIGHT AFFECT RESEARCH, PRACTICE OR POLICY**

- Practice implications of the evidence gap are that neurodiversity inclusive practices are not evidence informed in sport.
- The needs of neurodivergent athletes may be overlooked and the environmental, social, and emotional needs of neurodivergent athletes are yet to be defined and identified in the scientific literature.

## Introduction

Mental health in elite sport is increasingly recognised as a foundation for performance and well-being of athletes,^1–3^ however, there is little evidence summarising the state of evidence in relation to neurodiversity in this unique setting. Neurodiversity affirming practice is a strengths-based approach to education and ability.^4 5^ The term neurodiversity has emerged in response to the potential stigmatisation of medical diagnoses, and the recognition of the need for social models of disability and strengths-based approaches in understanding experiences and assets of neurodivergent groups. There is debate in the literature as to the appropriate language used for referring to neurodivergent populations.^6 7^ While medical and scientific experts have historically viewed person-first language (ie, person with autism) as most appropriate, this has been opposed by many individuals in the autism community who advocate identity-first language as least harmful (ie, autistic person).^6^ Research supports the preferred use of identity-first language for a proportion of individuals.^8^ Both person-first and identity-first language are used throughout this work to reflect the diversity in preference regarding language.^7 9^

Neurodiversity assumes there are variations in cognition, social learning and other behaviours which are normal variations in the context of human neurodevelopment. It is recognised there is ongoing discussion among academic, community and practitioner communities relating to consensus in the definition of neurodiversity, neurodivergence and neurotypicality.^10–12^ For this systematic scoping review, neurodiversity refers to the broad range of cognitive, behavioural, social and emotional presentations across individuals. Neurodivergence is used as a broad term incorporating autism spectrum disorder, attention-deficit hyperactivity disorder (ADHD) and other neurodevelopmental conditions including specific learning disorders. It is also accepted that heterogeneity exists in presentations of neurodivergence, and that aggregating evidence must be in the context of such heterogeneity.^12 13^ As per the American Psychiatric Association Diagnostic and Statistical Manual of Mental Disorders, ADHD is characterised by differences of inattention and/or hyperactivity impulsivity that interferes with functioning or development.^14^ Autism is characterised as social communication differences, and restricted, repetitive and/or sensory behaviours or interests. ADHD and autism have been overlooked in the broader context of elite athlete mental health,^15^ and there is a broader need to understand the unique experiences of neurodiversity.^16^

It has been estimated that the prevalence of autism is approximately 0.70%–3% in those under 18 years, although prevalence estimates vary.^17^ Approximately 5%–11% of those under the age of 18 years have been diagnosed with ADHD.^17^ Specific learning disorders have been proposed to occur at about 3%–10% of the global population.^17^ The historical context of neurodevelopmental conditions is important to consider when assessing prevalence of neurodivergence. As an example, diagnostic criteria have traditionally overlooked the specific characteristics for women and girls such as masking, leading to very large proportions (estimated around 80% in some studies) to be misdiagnosed.^18 19^ Masking or camouflaging, whereby neurodivergent individuals adopt neurotypical traits to adapt to environmental or other challenges, is a known concept through which neurodivergence can go under-recognised.^20^ Masking is a social compensation or coping strategy and may be used in environments where neurodivergent behaviours are not accepted or understood in order to belong or avoid stigma or bullying.^21^ The cognitive and emotional efforts of masking have been associated with stress, anxiety, depression and exhaustion.^22 23^

Elite sport is a setting of interest given the increasing understanding and importance of mental health and well-being among athletes, and the extant evidence to date in the field.^3 24 25^ There has been suggestion that the demands of elite sport, such as intense focus and structure for training regimes, high energy expenditure, structured play and particular personality and cognitive requirements and strengths, may appeal particularly to the unique abilities of neurodivergent groups, respectfully noting that heterogeneity that exists within neurodivergent groups.^26^ As an example, the organised way in which social interaction occurs in sport may be protective for those with social-emotional reciprocity differences, enhanced by the predictability of peer socialisation within sport. Conversely, there are known behavioural, cognitive, sensory and other needs of neurodivergent individuals that may be exacerbated in the elite sport setting.

The research relating to best practice regarding mental health and well-being in elite sport is rapidly evolving, such as with the publication of the International Olympic Committee Consensus Paper on mental health in elite athletes, and the promotion of best practice frameworks for prevention and promotion in elite sport settings.^24 25^ Given the large proportion of neurodivergent individuals across the general population and the unique stressors and characteristics of elite sport, it is important that the state of neurodiversity in elite sport is assessed. This will allow the needs of these individuals to be incorporated into future best practice so that sporting environments adapt to meet the needs of neurodivergent athletes and the health and well-being of neurodivergent athletes is understood and supported by sporting organisations.

Overall, this systematic scoping review aimed to understand the current state of knowledge in the elite sport field in relation to neurodiversity, including what key characteristics or factors relating to neurodiversity in elite sport have been identified, and in turn, to establish the knowledge gaps.

## Methods

### Protocol and registration

This systematic scoping review was informed by Preferred Reporting Items for Systematic Reviews and Meta-Analyses (PRISMA) Extension for Systematic Scoping Reviews (checklist attached as online supplemental table 1). This systematic scoping review was prospectively registered on Open Science Framework on 12 August 2022 (osf.io/36w7m).

10.1136/bmjsem-2023-001575.supp1 Supplementary data

### Eligibility criteria

Studies were eligible for review if they examined neurodivergence in elite sport. Neurodivergence included autism, ADHD and learning disorders including dyslexia, dyscalculia and dysgraphia (now known as specific learning disorders). Studies that considered other recognised neurodiversity areas of interest (eg, Tourette syndrome) were eligible for review. Elite sport was defined as Olympic, Paralympic, national, international, professional and semiprofessional athletes and the systems in which they train and compete (ie, elite coaching, elite sport leadership, professional sporting organisational research were eligible for review). Study types eligible for review included observational, intervention, perspective or commentary studies and reviews. Case studies and reviews of case studies were ineligible for review, as was grey literature. Exclusion criteria were studies that focused on sport participants not in elite categories as above (ie, community level, general population), non-English language publications, and studies that included neurodiversity in the aims and objectives but failed to report on outcomes relating to neurodiversity (eg, one study included ADHD in search terms but did not report on findings related to neurodiversity and was therefore excluded).^27^

### Search strategy

**Figure 1:**
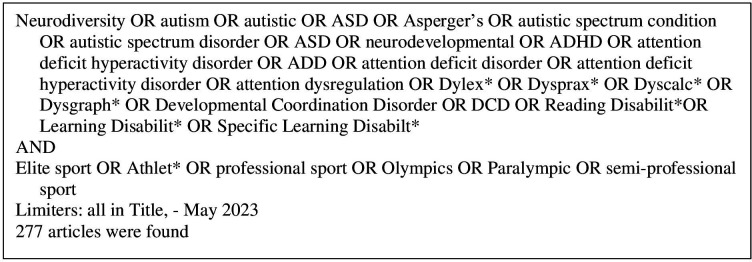
Search strategy.

The following databases were searched to identify all relevant literature; Academic Search Complete, CINAHL Complete, Health Source—Nursing and Academic Edition, MEDLINE Complete, APA PsycINFO and SportDISCUS with full text, all accessed via EBSCOHost. Keywords were related to neurodiversity, autism, ADHD, neurodevelopmental and elite sport key words (figure 1). All English language publications since journal inception until 12 May 2023 that met the above criteria were eligible for review. Figure 1 describes the search strategy used in this systematic scoping review. We extended this search strategy to examine all reference lists of identified studies to ensure all relevant literature was sourced. We also examined all included studies within identified literature reviews and used this strategy to allow iterative searches to identify any further possible literature (ie, if a study appeared relevant the reference list of that particular study was also screened).

**Figure 2:**
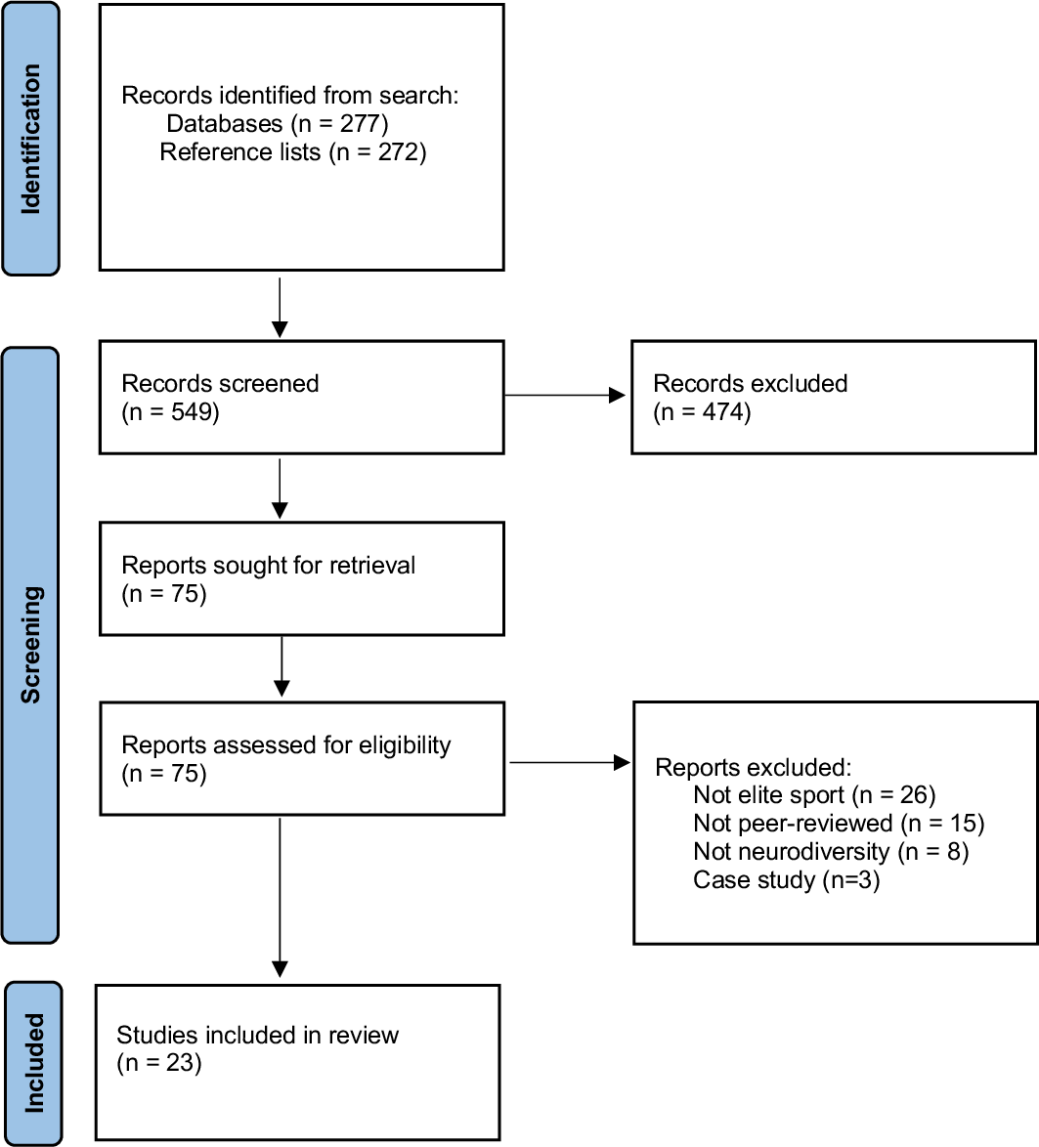
PRISMA flow chart. PRISMA, Preferred Reporting Items for Systematic Reviews and Meta-Analyses.

Title and abstract screening were completed by one author (EH), following which full text screening occurred with articles that appeared eligible. A second reviewer screened 10% of articles to ensure consistency (JR). A third reviewer (LO) was consulted for discrepancies. Reference lists of identified reviews were screened to identify any further possible articles eligible for review. PRISMA flow chart for article selection is presented in figure 2.

### Data items

A data extraction tool was developed which extrapolated data on study author, year, country, type of evidence (observational, review, longitudinal, experimental, qualitative, viewpoint/commentary, other), study aims, participants, findings and overall implications for the study of neurodiversity in elite sport settings. The data extraction tool was used to report the summary of results in online supplemental table 2.

10.1136/bmjsem-2023-001575.supp2 Supplementary data

## Results

### Study characteristics

A summary of studies reviewed is presented in table 1. Online supplemental table 2 describes overall findings. Of the 277 articles identified in the above search, and 272 articles screened through reference lists of identified studies, there were 76 that met criteria for full-text review. On full-text screening, there were 23 studies that were identified as eligible and were subsequently included in this review. Online supplemental table 3 reports on the excluded articles and reasons for exclusion. Generally, articles were excluded due to not being elite sport focused (eg, community-level, school-level sport) and not focusing on neurodivergence (eg, neuropsychological assessment in the general population) (online supplemental file 3). Most of the reviewed literature was published in the last decade since 2012. One study examined specific learning disorders.^28^

10.1136/bmjsem-2023-001575.supp3 Supplementary data

**Table 1:** Summary of studies included in the review

Author, year	Study type	Focus	Sources	Implications
Åkesdotter, 2020^29^	Observational	ADHD	College athletes	Prevalence
Alosco, 2014^31^	Observational	ADHD	College athletes	Concussion screening, treatment, management
Beidler, 2021^32^	Observational	ADHD	College athletes	Concussion screening, treatment, management
David, 2022^33^	Observational	ADHD	Professional footballers	Concussion screening, treatment, management
Ekman, 2021^30^	Observational	ADHD	Youth athletes	Prevalence
Gunn, 2022^28^	Observational	ADHD	College athletes	Concussion screening, treatment, management
Manderino, 2018^35^	Observational	ADHD	College athletes	Concussion screening, treatment, management
Manderino, 2019^36^	Observational	ADHD	College athletes	Concussion screening, treatment, management
Nelson, 2016^37^	Observational	ADHD	College athletes	Concussion screening, treatment, management
Li, 2021^34^	Observational	ADHD	College athletes	Anxiety management
Kutcher, 2011^38^	Review	ADHD	Published literature	Medical treatment
Stewman, 2018^39^	Review	ADHD	Published literature	Prevalence
Han, 2019^26^	Review	ADHD	Published literature	Prevalence and treatment
White, 2015^40^	Review	ADHD	Published literature	Sport performance
Garner, 2018^41^	Commentary	ADHD	Published literature and professional expertise	Treatment
Parr, 2011^42^	Commentary	ADHD	Published literature and professional expertise	Lived experience and treatment
Putukian, 2011^43^	Position statement	ADHD	Published literature and professional expertise	Treatment and management
Pujalte, 2023^45^	Position statement	ADHD	Published literature and professional expertise	Diagnosis and management
Reardon, 2016^44^	Commentary	ADHD	Published literature and professional expertise	Management
Ciocca, 2019^46^	Commentary	ADHD	Published literature and professional expertise	Treatment
Duquesne, 2022^48^	Qualitative	Autism	Elite table tennis players and track and field athletes	Lived experience
Palmer, 2003^49^	Qualitative	ADHD	College athletes	Lived experience
Cushing, 2020^47^	Qualitative	ADHD	College athletes	Lived experience, treatment and management

ADHD, attention-deficit hyperactivity disorder.

The most frequent study design was observational with 10 studies (48%) examining athlete cohorts through surveys, medical examination data or other routinely collected information regarding athlete health and well-being.^28–37^ Of the observational studies, six were conducted in the USA,^28 31 32 35–37^ one was conducted in Canada,^33^ two in Sweden,^29 30^ one in China.^34^ The remaining studies were systematic or narrative reviews,^26 38–40^ commentary/position statements^41–46^ and qualitative studies,^47–49^ conducted in the USA^47 49^ and France.^48^ In terms of populations studied within the review literature, most work focused on college athlete groups, primarily in the USA.^28 31 32 35–37 47 49^ Professional Canadian footballers were studied in one reviewed publication.^33^ One study focused on Swedish national athletes applying for university sporting scholarships.^29^ Another study examined Swedish youth who were enrolled in the Swedish national sports talent programme.^30^ French elite table tennis and track and field athletes were studied in another included paper.^48^

### Findings in reviewed studies

#### Concussion

The literature to date on neurodivergence in elite sport has largely focused on ADHD, and specifically on the screening, treatment and management of concussion among elite athletes with ADHD.^28 31–33 35–37^ Among the college athlete population, ADHD has been examined as a risk factor for previous and future concussion. The diagnosis of ADHD has been shown to impact baseline screening performance on concussion assessments and was further implicated in the subsequent treatment and management outcomes following a concussion.^31 32 36^ ADHD appears to co-occur with greater rates of single and multiple concussions, and the validity of concussion assessments may differ among those with ADHD compared with neurotypical peers.^35 36^

#### Prevalence

Prevalence studies suggest the elite athlete population may present with ADHD at greater rates than that observed at the general population level.^26 29 30 39^ There is suggestion that ADHD may provide competitive advantage in elite sport, such as rapid reaction and attention diversion to random emerging stimuli.^50^ There was evidence for this finding earlier in life: one study examined whether ADHD was more present among youth athletes in the national Swedish sports talent programme, compared with non-athletes, finding that athletes displayed greater alignment with ADHD criteria.^30^ Interestingly, this study found that the ADHD criteria were observed at a higher level in the education setting for athletes, compared with the sporting environment.^30^

#### Treatment

There has also been a focus on the safe and effective medical treatment of athletes with ADHD.^26 38 41 43 45–47^ Specifically, this body of research has examined the role of stimulants in medicating ADHD symptoms, and the potential to conflict with sport doping codes. Indeed, research identified through this review examined the need for appropriate treatment strategies for ADHD, in the context of high performance and ensuring athlete health and well-being generally.^40 42 45 46^ A recent position statement from the American Medical Society for Sports Medicine highlighted the need for physicians to strive for early diagnosis, multidisciplinary supports and other supports to allow athletes to successfully compete in elite sport.^45^

### Qualitative research

There were three studies that considered the qualitative lived experience of autistic athletes and athletes with ADHD.^47–49^ The focus of the qualitative studies, all conducted with elite athletes through interviews, was to understand the lived experience of neurodivergence in elite sport, and specifically how their diagnosis impacts their ability to engage in high-performance sporting settings. They considered whether there were specific barriers to excelling in elite sport, relative to living with ADHD and autism. Just one study of the included 23 papers focused on autism in elite sport, which specifically examined the lived experience of autistic French elite table tennis players and track and field athletes through qualitative interview.^48^ Findings across qualitative studies suggested the need for sporting organisations to ensure flexibility to meet the needs of neurodivergent athletes. Athletes identified a range of challenges including lack of understanding of neurodiversity in sporting contexts, coping mechanisms associated with diagnosis and positive benefits of being engaged in sports such as inclusion.

## Discussion

The findings of this systematic scoping review indicate that while neurodivergence is likely to be present in elite sport there is very limited research in this space to date. Most studies identified through this review focused exclusively on ADHD in college athlete populations based in the United States. There was a specific focus on the role of ADHD as a risk factor for concussion, and the subsequent management of athletes with ADHD in concussion screening, treatment and management. There was also a focus on the appropriate treatment and management of symptoms of ADHD in athlete populations. Specifically, these studies considered the appropriateness of stimulant medications for the treatment of ADHD symptoms among athletes, and specifically considered in the context of adherence to sporting doping codes to which stimulants are typically banned. A smaller body of research focused on the lived experience of athletes, identifying that the unique experiences of athletes with ADHD and autism warrant careful consideration in terms of providing tailored supports to athletes for health, performance and well-being. Most of the literature to date has examined college athlete populations, based in the USA. Just one study examined autism in elite sport, examining the lived experiences of French elite table tennis and track and field athletes. Notably, there were no studies of sports adopting practices or approaches informed by an understanding of the social, cognitive and environmental needs of neurodivergent athletes.

### Findings in the context of existing knowledge

The lack of literature examining neurodivergence in elite sport reflects the dearth in research examining mental health and well-being generally in athlete populations. There have been recent calls to action to establish frameworks to support athlete mental well-being, that go beyond building mental health literacy, to providing systems that support and enable athletes to reach their athletic and non-athletic potential.^24 51^ There are additional sport-specific risks (eg, media scrutiny, performance and training pressures) that athletes experience that may place them at risk for poorer mental health outcomes.^3^ There is ongoing need to further understand the prevalence, experiences and outcomes of elite athletes, specifically regarding mental health and, as identified in this review, among neurodivergent populations.

Research to date has focused on the association between concussion and clinical outcomes among individuals with ADHD. Among community-level athletes, premorbid ADHD is found to be associated with greater symptom report,^52^ and worse cognitive performance at baseline assessment,^53^ as well as a greater prevalence of having a previous concussion^52^ and a greater propensity to incur concussion within the sporting season.^54^ Findings regarding protracted recovery following sports-related concussion, however, remain mixed^52 55^ possibly due to the lack of research with well controlled methodology. In a systematic review by Cook *et al* including 14 studies constituting 3623 participants (n=359 (9.9%) with ADHD), the authors found only two studies with a statistical association between ADHD and prolonged recovery—with both studies having limited sampling (21 participants total).^56^ Of note, only one other study in the review was designed specifically to examine ADHD and protracted recovery post sports-related concussion without any significant findings.^56^

Interestingly, it has been suggested that ADHD is more prevalent in athlete populations than non-athlete populations^26 29 30 45 46^ although prevalence data to confirm this are limited. Pagani *et al* argued that the demands of competitive sport (eg, intense dedication, structured regimens, high physical stamina requirements) may have a protective role for individual attributes of people with ADHD (eg, need for energy outlet, need for routine, hyperfocus, high energy stores) thereby increasing the prevalence of ADHD in sport disproportionally to the population.^57^ It is also possible that athletes with ADHD are more likely to succeed in terms of performance in elite sport given the inherent traits and characteristics of ADHD. Hyperfocus and rapid reaction to changing stimuli has been reported to occur among people with ADHD when there is an immediate source of feedback, and when the individual is highly stimulated and can ignore other distractions.^58^ It is conceivable that these skills are likely to be highly advantageous in elite sport settings which require rigorous training and focus, structured skill development, responding to feedback and other goal orientated behaviours. Conversely, it is also likely that there may be limiting aspects to neurodivergence in the context of elite sport such as uncertainty and changing environments, which may pose unique challenges to such groups.^59 60^ Ultimately, further research is needed to explore such experiences, recognising the heterogeneity that exists within neurodivergent groups which requires research centred on lived experiences and community participatory action research in which researchers and community members are equal partners.

It is of interest that there was so little research examining autism in elite sport, given the high prevalence reported globally and the high co-occurrence rate with ADHD.^61^ Indeed, much of the excluded literature in this current review related to autism in sport, although at the community and school levels. There is likely improved psychological well-being, cognitive and physical health outcomes among autistic individuals as a result of physical activity and sport participation.^62^ The historical context of autism is relevant to this current review in that there is evidence to suggest high rates of misdiagnosis, and in particular misdiagnosis among women, and among adult presentations of autism.^63 64^ For example, diagnostic processes have been refined and altered corresponding to improved understandings of autism presentations among women, who have historically been overlooked in autism diagnostic criteria.^65^ It is, therefore, possible that the lack of evidence to date examining autism in athlete populations relates to historical poor diagnostic understanding, reduced access to experienced professionals and through other socio-cultural mechanisms such as stigma at the general population level. In the general community, an autistic individual may develop masking techniques and other coping mechanisms to manage their symptoms in adult and later life.^20^ The consequences of masking behaviours with anxiety, stress, depression and lifetime suicidality have been established.^66^ Indeed, common mental disorders are highly prevalent in older populations who have misdiagnosed autism.^67^ Taken together, the current findings identify a current unmet need in understanding autism in the elite sport setting.

### Gaps in evidence

The available literature regarding neurodivergence in elite sport is limited, and of the evidence that does exist, this relates almost exclusively to concussion experienced by ADHD athletes, and the medical treatment of ADHD in the context of antidoping regulations. There is some evidence relating to the lived experience of neurodivergent athletes, but again this mostly related to ADHD. There was a gap in evidence in autism in elite sport across all study types. Further, while there were some prevalence, treatment and management studies (at least for ADHD), there were no identified intervention or clinical practice focused studies on supporting the neurodivergent athletes regarding specific needs.

Notably, there were no studies focused on environmental practices to support adaptions in the elite sport setting for neurodivergent needs. This is inconsistent with present ethos in community and education which advocates for environmental adaptions in line with strengths-based approaches for neurodiversity inclusivity.^11 68^ As a practical example, education of coaches and performance staff around sensory, emotional and cognitive needs of neurodivergent athletes is required. Healthcare staff such as sports physicians, physiotherapists and other professionals would likely benefit from such education. This was a particularly important finding given the shift towards psychological safety in sport which values the individuality of athletes, and the recommended systemic approaches to athlete well-being which promote cultures of inclusivity.^24 69^ It can be surmised the needs of autistic athletes may be overlooked and the environmental, social and emotional needs of neurodivergent athletes are yet to be defined and identified in the scientific literature. Practice implications of this evidence gap are that evidence-based neurodiversity inclusive practices are limited in elite sport.

It remains unclear whether ADHD, for example, confers any advantages to elite athletes, although literature has suggested this is possible.^45^ It is conceivable that hyperactivity in a classroom may be commonly observed as a disadvantage, whereas the sports field these skills may be an advantage. The same might apply to increased reactivity to ambient stimuli. Autism can confer deep interest and focus on singular topics that could be perceived to be disadvantage in a social setting, but possibly an advantage in a domain requiring singular focus and diligence.^70^ The above considerations are yet to be explored in the elite sport literature to date but are warranted given the potential for strengths-based approaches.^71^ There is also the consideration that the significant transitions experienced in elite sport, such as the transition to postcareer retirement, may hold unique implications for neurodivergent individuals. This further justifies the need for future research in this area.

There were methodological limitations evident across the reviewed studies that affected interpretation of findings. This included self-report measures of neurodevelopmental differences which are amenable to individual bias compared with clinician diagnostic interviews and other more objective measures. Sample sizes were often small, and this limits the generalisability of findings, particularly in the context of elite sport which is highly heterogeneous across sporting codes. Our search strategy for this scoping review was pragmatic and iterative in its approach and it is possible that literature was overlooked due to the methods adopted (ie, the inclusion of abstract in the search strategy limiters may have allowed more comprehensive literature search and may have led to further evidence available for review). We propose this scoping review as an important summary of evidence to date, but do not assume that the studies included in this review are exhaustive. As a consequence, our scoping review did not examine grey literature and it is possible that we failed to incorporate relevant evidence. A limitation of the evidence generally is the heterogeneity in the definition of athlete populations, with a large proportion of excluded literature being categorised as such due to focusing on sport participation in the general community (online supplemental table 3). Finally, given the wider movement and advocacy among neurodivergent populations in terms of research and practice, it is important to note that there was little evidence for research adopting strengths-based approaches for neurodiversity(eg, well-being supports for athletes to accommodate unique neurodivergent needs) in the elite sport setting.

## Conclusion

This systematic scoping review found limited evidence examining neurodivergence in elite sport, despite suggestions that ADHD may occur at higher rates in athlete populations, the recognised connection between sport performance and supporting the mental health and well-being needs of athletes, and the high prevalence of neurodivergence in the wider population. There is a strong rationale for future research to build on this evidence, given the younger age at which individuals often first commence their pathways into elite sport coincides with the developmental period during which ADHD, autism and other neurodevelopmental differences are first identified. There are performance and coaching-related reasons for understanding neurodiversity in elite sport, particularly given the learning and developmental processes involved in acquiring and refining skills that are required to succeed in competition. Finally, there are known unique social, emotional, cognitive, behavioural, sensory and other needs that correspond to neurodiversity, and to truly create supportive elite sporting systems it is crucial such needs are understood and addressed in this context. As such, there is a need for evidenced-informed approaches that achieve as such, and therefore, an understanding of the sociocultural contexts as they apply in sport are urgently needed as a future focus of research.
