# Supplementary material for: Neurodiversity in elite sport: a systematic scoping review
Source: BMJ Open Sport Exerc Med. 2023 Jun 15;9(2):e001575. doi: 10.1136/bmjsem-2023-001575 (PMC10277550; doi:10.1136/bmjsem-2023-001575)
Supplement: Supplementary data [file bmjsem-2023-001575supp002.pdf]

Supplementary File 2

Table of results

Author, Year, Country	Type of evidence	Study aims	Participants, Methods	Findings
Åkesdotter et al., 2020<br><br>Sweden	Observational	To examine mental health problems in Swedish national elite athletes.	333 National Swedish athletes across multiple sports applying for University scholarships.	Symptoms of ADHD were found to be 5.4% overall, and 5.1% in females and 5.8% in males.
Alosco et al., 2014<br><br>United States	Observational	To examine the link between ADHD and concussion risk among athletes.	139 NCAA Division-I athletes self-reported a history of prior concussion and diagnostic history of ADHD.	ADHD was found in 10% of the sample and one fifth of the sample reported a prior history of concussion. Half of athletes with ADHD reported a history of at least one prior concussion compared to 14.4% of athletes without ADHD.
Beidler et al., 2021<br><br>United States	Observational	To examine whether ADHD diagnosis in collegiate athletes was associated with diagnosed and nondisclosed concussions, and reasons why suspected concussive injuries were not disclosed.	858 collegiate athletes (65 with ADHD; 793 without ADHD). The survey included self-report items regarding ADHD status and diagnosed and nondisclosed sport-related concussions.	ADHD was associated with greater likelihood of reporting concussions.
David et al., 2022<br><br>Canada	Observational	Canadian Football League athletes were assessed in regards to ADHD status and the relationship with concussion.	784 footballers completed mental health measures including ADHD, physical functioning, depression, cognitive functioning.	Physical Functioning, Depression and Cognitive domains differed between ADHD athletes and non-ADHD.
Ekman et al., 2021	Observational	To explore the symptoms of ADHD in young athletes compared to non-athletes	200 high school students, 40 women and 69 men in the athlete group (students enrolled in the Swedish national sports talent program) completed the ADHD Self-report Scale.	ADHD criteria differences were found for athletes during school time compared to sports time.
Gunn et al., 2022<br><br>United States	Observational	To determine the risk of concussion in athletes with ADHD, learning disability, or both.	Data from the Concussion Assessment, Research and Education (CARE) Consortium (2014–2017) were used to evaluate concussion in athletes with ADHD,	Athletes with self-reported diagnosis of ADHD, LD, and ADHD+LD were more likely to report a single concussion and multiple concussions.

			learning disability, or both, relative to controls.	
Manderino et al., 2018<br><br>United States	Observational	To determine whether student athletes with history of ADHD or learning difficulties produce lower concussion risk scores and are more likely to produce invalid protocols than neurotypical athletes.	949 NCAA athletes with. 6.8% ADHD, 5.6% Academic Difficulties, 2.0% comorbid ADHD/Academic Difficulties.	Student athletes in the academic difficulties and comorbid groups performed worse on concussion risk scores, but not for those only with ADHD.
Manderino et al., 2019<br><br>United States	Observational	To determine whether performance on concussion screening protocols differs for athletes with and without ADHD.	2086 NCAA athletes were assessed in regards to concussion risk profiling.	Athletes with ADHD and learning difficulties were more likely to produce invalid proto-cols.
Nelson et al., 2016<br><br>United States	Observational	To determine how ADHD and learning difficulties are associated with concussion history and performance on standard concussion assessment measures.	The study sample aggregated data from two separate projects: the National Collegiate Athletic Association Concussion Study and Project Sideline.	ADHD was associated with prevalence of multiple historical concussions, greater baseline symptoms, and poorer performance on cognitive assessments..
Li et al., 2021<br><br>China	Observational	The aim of this study was to examine the prevalence of anxiety disorder and its risk and protective factors in elite collegiate athletes.	285 college athletes from China completed a self-report form assessing GAD and potential predictors including attention deficit hyperactivity disorder (ADHD).	ADHD was found to be associated with increased risk of anxiety.
Kutcher et al., 2011<br><br>NA	Review	This review reports on a. framework for the treatment of ADHD among athletes.	Not reported.	ADHD medication is of particular interest given the social and other impacted functioning and the potential bans due to doping codes.
Stewman et al., 2018<br><br>*NA*	Review	This review aimed to synthesise literature to determine how common ADHD is in the athlete population.	Literature searches were performed on databases for the years 2000 to 2016 utilizing the following key search terms: ADHD, ADD, guidelines, diagnosis, athlete, sports, treatment, pharmacotherapy, stimulants, risk, cardiovascular effects, concussion, and traumatic brain injury.	ADHD exists among athletes at all levels, and there is overlap with concussion presentation.

Han et al., 2019<br><br>NA	Review	This review aimed to report on prevalence, symptoms, and treatments of ADHD in elite athletes.		The symptoms and characteristics of ADHD may impact choice of sport and level of eliteness achieved. Management of ADHD in elite athletes is important for safety and performance, and must be considered in the context of doping codes.
White et al., 2015<br><br>NA	Review	To review literature on ADHD and elite athletes.	Studies were selected through literature searches of PubMed, MEDLINE, and Cochrane databases for the years 1991 to 2011. Key search terms were ADD, ADHD, sports, athletes, athletics, elite sport and injury associated terms including concussion.	ADHD usually has an early onset, with delayed diagnosis in some patients due to heterogeneous presentations.
Garner et al. 2018<br><br>NA	Commentary	The commentary aims to address common concerns raised about stimulant use in elite sport, highlighting current gaps in evidence in regards to banning stimulants for athletes with ADHD.	NA	The findings indicated that banning stimulant use among athletes with ADHD does not support fair play, ensure safety, or align with existing policies of large governing bodies. The authors argue that in high-level sport, tailored and appropriate support should be given.
Parr et al., 2011<br><br>NA	Commentary	This commentary discusses the experience of elite sport for athletes with ADHD.	NA	The incidence of ADHD may be increased among athletes who participate at the elite level. Neurotransmitters involved in ADHD mean that medications used to treat ADHD may affect performance.
Ciocca, 2019<br><br>NA	Commentary	This commentary discusses ADHD in athletes including considerations for treatment and management.	NA	Objective assessment of ADHD makes prevalence unclear. Treatment considerations include sport drug governing bodies.
Putukian et al., 2011<br><br>NA	Position statement	To provide a statement on ADHD in elite sport.	NA	Physicians should understand the side effects of medications, regulatory issues regarding stimulant medications, and indications for additional testing in order to appropriately support athletes with ADHD.
Pujalte et al., 2023<br><br>NA	Position statement	To provide a position statement on ADHD in athletes, specifically in	Systematic literature search and professional expertise.	Those working with athletes with ADHD should strive to provide high quality of care. This includes early diagnosis, appropriate and careful

		regards to diagnosis, treatment and management on behalf of the American Medical Society for Sports Medicine,		multidisciplinary treatment, and complete and timely documentation to facilitate continued sports participation.
Reardon et al., 2016<br><br>NA	Commentary	This commentary discusses the medical management of athletes with ADHD given it is a controversial issue.	NA	The issues related to stimulant medications in athletes are multi-faceted. Some proposals are made regarding the use of stimulants by athletes.
Duquesne et al., 2022<br><br>France	Qualitative	The aim of this study was to understand the experiences of elite athletes with intellectual disabilities (ID) and/or autism spectrum disorders (ASD).	Fifteen semi-structured interviews and eight days of ethnographic observations were carried out with 7 elite table tennis players and 8 elite track-and-field athletes.	There was heterogeneity in experiences - positive and negative - of elite ID and/or ASD athletes. There is evidence to support elite sporting organisations ensure flexibility.
Palmer, 2003<br><br>United States	Qualitative	This qualitative study aimed to understand college student-athletes' experience of living with attention deficit hyperactivity disorder.	Interviews with 6 Division I college student-athletes with ADHD were completed.	Major themes included academic challenges, medication issues, difficulty balancing, lack of understanding by others, a belief in the unfairness of having the disorder, a belief of not being normal, the buoyancy effect of sport, and self-monitoring/internal dialogues.
Cushing, 2020<br><br>United States	Qualitative	The purpose of this study was to examine the psychological impact of being diagnosed and treated for ADHD among NCAA division-1 athletes.	8 current and former student-athletes (aged 20–32) of various sports were interviewed.	Findings revealed challenges associated with the pressure of excelling in both sport and academics, as well as increasing knowledge of the coping mechanisms associated with such a diagnosis.
