# Supplementary material for: Neurodiversity in elite sport: a systematic scoping review
Source: BMJ Open Sport Exerc Med. 2023 Jun 15;9(2):e001575. doi: 10.1136/bmjsem-2023-001575 (PMC10277550; doi:10.1136/bmjsem-2023-001575)
Supplement: Supplementary data [file bmjsem-2023-001575supp003.pdf]

Supplementary File 3

Excluded studies with reasons for exclusion

	Study title	Reason for exclusion
1	Gulick, C. N. (2011). Adverse Medication Response of an Elite Athlete With ADHD. *International Journal of Athletic Therapy and Training*, *16*(3), 41-44.	Not a peer-reviewed publication.
2	Brown, T. E. (2017). *Outside the box: rethinking ADD/ADHD in children and adults: a practical guide*. American Psychiatric Pub.	Not a peer-reviewed publication.
3	A Can-Do Approach to Autistic Children and Athletics	Not a peer-reviewed publication.
4	Barak, S., Oz, M., Dagan, N., & Hutzler, Y. (2019). The Game of Life soccer program: Effect on skills, physical fitness and mobility in persons with intellectual disability and autism spectrum disorder. *Journal of Applied Research in Intellectual Disabilities*, *32*(6), 1401-1411.	Not elite athlete
5	Champ, F. (2018). *Psychological development in professional youth football: An ethnography of sports psychology practice*. Liverpool John Moores University (United Kingdom).	Not neurodiversity focused.
6	Cruickshank, A., Collins, D., & Minten, S. (2015). Driving and sustaining culture change in professional sport performance teams: A grounded theory. *Psychology of Sport and Exercise*, *20*, 40-50.	Not neurodiversity focused.
7	Denning, C. B., & Ghio, K. (2014). Impact of the Young Athletes Program on Young Children with Autism Spectrum Disorders in Quincy Public Schools.	Not elite sport focused.
8	Dove, J., & Rowe, L. (2020). Cycling: Attention Deficit Hyperactivity Disorder and Anti-Doping. *Case Studies in Sports Psychiatry*, 54	Book chapter.
9	Milewski, M. D., Skaggs, D. L., Bishop, G. A., Pace, J. L., Ibrahim, D. A., Wren, T. A., & Barzdukas, A. (2014). Chronic lack of sleep is associated with increased sports injuries in adolescent athletes. *Journal of Pediatric Orthopaedics*, *34*(2), 129-133.	Not elite sport.
10	Tod, D., Hutter, R. V., & Eubank, M. (2017). Professional development for sport psychology	Book chapter

	practice. *Current opinion in psychology*, *16*, 134-137.	
11	Jimeno, M. (2019). Improving the quality of life of children and adolescents with autism spectrum disorders through athletic-based therapy programs. *The Educational and Developmental Psychologist*, *36*(2), 68-74.	Not elite sport
12	Jones, P., Derby, K. M., Engler, J. R., & Trotter, T. (2019). Training of Novice Trainers to Work with Athletes with Autism Spectrum Disorders in an Ice Hockey Practice Setting. *Insights into Learning Disabilities*, *16*(2), 123-137.	Not elite sport.
13	Klenck, C., & Gebke, K. (2007). Practical management: common medical problems in disabled athletes. *Clinical Journal of Sport Medicine*, *17*(1), 55-60.	Not ND focused.
14	Luna, L. D. (2020). *The Perceptions of Academically Underachieving, Gifted, Elite Male Athletes: A Grid and Group Interpretation* (Doctoral dissertation, Aurora University).	Not ND focused.
15	Martin, G. L., Vause, T., & Schwartzman, L. (2005). Experimental studies of psychological interventions with athletes in competitions: Why so few?. *Behavior modification*, *29*(4), 616-641.	Not ND focused.
16	Meyer, J., & Arnett, P. A. (2019). Do traditional neuropsychological measures add value to computerized tests for concussion assessment in collegiate athletes?.	Not ND focused.
17	Patatoukas, D., Farmakides, A., Aggeli, V., Fotaki, S., Tsibidakis, H., Mavrogenis, A. F., ... & Papagelopoulos, P. J. (2011). Disability-related injuries in athletes with disabilities. *Folia Med*, *53*(1), 40-6.	Not ND focused.
18	Ryan, S., Fraser-Thomas, J., & Weiss, J. (2018). Patterns of sport participation for youth with autism spectrum disorder and intellectual disabilities. *Journal of Applied Research in Intellectual Disabilities*, *31*(3), 369-378.	Not elite sport focused.
19	Van Biesen, D. (2021). Virtus Academy–driving the development of elite sport for athletes with intellectual impairment. *EUJAPA*, *14*(2), 8-8.	Not peer reviewed publication.

20	Wolfe, E. S. (2017). ADHD and Athletic Training: It Is Time for a Paradigm Shift in Patient Care and Cultural Norms. *The ADHD Report*, *25*(3), 8.	Not elite sport focused.
21	Zhang, K., Liu, Y., Liu, J., Liu, R., & Cao, C. (2021). Detecting structural and functional neuroplasticity in elite ice-skating athletes. *Human Movement Science*, *78*, 102795	Not ND focused.
22	Burns, J., & Johnston, M. (2020). Good Practice Guide for coaching athletes with Intellectual Disabilitie	Not peer reviewed publication.
23	Vorobyov, S. A., & Gretsov, A. G. (2022). Psychological preparation of coaches to work with athletes with autism spectrum disorders. *Theory and Practice of Physical Culture*, (5), 37-40.	Not peer reviewed.
24	Gulick, C. N. (2011). Adverse Medication Response of an Elite Athlete With ADHD. *International Journal of Athletic Therapy and Training*, *16*(3), 41-44.	Case study
25	Gulick, C. N. (2011). Adverse Medication Response of an Elite Athlete With ADHD. *International Journal of Athletic Therapy and Training*, *16*(3), 41-44.	Poster presentation.
26	Phrathep, D., Donohue, B., Kraus, S., Paul, M., & Mercer, J. (2022). A Controlled Evaluation of a Sport-Specific Performance Optimization Program in an Athlete Diagnosed With Attention Deficit Hyperactivity Disorder and Oppositional Defiant Disorder Within the Context of COVID-19. *Clinical Case Studies*, *21*(3), 209-234.	Case study
27	Alexander, M. G., Dummer, G. M., Smeltzer, A., & Denton, S. J. (2011). Developing the social skills of young adult Special Olympics athletes. *Education and Training in Autism and Developmental Disabilities*, 297-310	Multiple case study design
28	Alexander, M. G., & Gardin, F. A. (2017). Strategies for Athletic Trainers to Provide Effective Treatment to People With Autism Spectrum Disorder. *International Journal of Athletic Therapy and Training*, *22*(6), 12-16.	Not elite sport focused.
29	Ali, M., Dreher, N., Hannah, T., Li, A., Asghar, N., Spiera, Z., ... & Choudhri, T. (2021). Concussion incidence and recovery among youth	Not elite sport focused

	athletes with ADHD taking stimulant-based therapy. *Orthopaedic journal of sports medicine*, *9*(10), 23259671211032564.	
30	Cook, N. E., Huang, D., Silverberg, N., Maxwell, B., Zafonte, R., Berkner, P., & Iverson, G. L. (2016). Concussion-Like Symptom Reporting in High School Student Athletes with ADHD. *PM&R*, *8*, S156-S156.	Not elite sport focused
31	Cook, N. E., Huang, D. S., Silverberg, N. D., Brooks, B. L., Maxwell, B., Zafonte, R., ... & Iverson, G. L. (2017). Baseline cognitive test performance and concussion-like symptoms among adolescent athletes with ADHD: examining differences based on medication use. *The Clinical Neuropsychologist*, *31*(8), 1341-1352.	Not elite sport focused
32	Covassin, T., Elbin, R. J., Deitrick, J. M., & Whalen, D. J. (2013). Effects of attention deficit hyperactivity disorder on neurocognitive performance and symptoms in concussed athletes. *Athletic Training & Sports Health Care*, *5*(6), 254-260.	Not elite sport focused
33	David, C. V., Varkovetski, M., Wagner, R., Ree-Fedun, Q., Hansen, J., Arends, T., ... & Mrazik, M. (2022). A-12 Mental Health Outcomes for CFL Athletes with ADHD. *Archives of Clinical Neuropsychology*, *37*(5), 1053-1053.	Conference abstract
34	David, C. V., Varkovetski, M., Wagner, R., Ree-Fedun, Q., Hansen, J., Arends, T., ... & Mrazik, M. (2022). A-12 Mental Health Outcomes for CFL Athletes with ADHD. *Archives of Clinical Neuropsychology*, *37*(5), 1053-1053.	Not elite sport focused
35	Iaccarino, M. A., Fitzgerald, M., Pulli, A., Woodworth, K. Y., Spencer, T. J., Zafonte, R., & Biederman, J. (2018). Sport concussion and attention deficit hyperactivity disorder in student athletes: A cohort study. *Neurology: Clinical Practice*, *8*(5), 403-411.	Not elite sport focused
36	Iverson, G. L., Atkins, J. E., Zafonte, R., & Berkner, P. D. (2016). Concussion history in adolescent athletes with attention-deficit hyperactivity disorder. *Journal of neurotrauma*, *33*(23), 2077-2080.	Not elite sport focused

37	Iverson, G. L., Wojtowicz, M., Brooks, B. L., Maxwell, B. A., Atkins, J. E., Zafonte, R., & Berkner, P. D. (2020). High school athletes with ADHD and learning difficulties have a greater lifetime concussion history. *Journal of attention disorders*, *24*(8), 1095-1101	Not elite sport focused
38	Kelly, C. A., Ketcham, C. J., Patel, K., & Hall, E. E. (2018). Test Setting and ADHD Influence Baseline Concussion Testing Neurocognitive Performance in Collegiate Student-Athletes: 2029 Board# 9. *Medicine & Science in Sports & Exercise*, *50*(5S), 495.	Not peer-reviewed publication.
39	Maietta, J. E., Barchard, K. A., Kuwabara, H. C., Donohue, B. D., Ross, S. R., Kinsora, T. F., & Allen, D. N. (2021). Influence of special education, ADHD, autism, and learning disorders on ImPACT validity scores in high school athletes. *Journal of the International Neuropsychological Society*, *27*(5), 461-471.	Not elite sport focused
40	Maietta, J. E., Kuwabara, H. C., Cross, C. L., Flood, S. M., Kinsora, T. F., Ross, S. R., & Allen, D. N. (2021). Influence of autism and other neurodevelopmental disorders on cognitive and symptom profiles: Considerations for baseline sport concussion assessment. *Archives of Clinical Neuropsychology*, *36*(8), 1438-1449.	Not elite sport focused
41	Mautner, K., Sussman, W. I., Axtman, M., Al-Farsi, Y., & Al-Adawi, S. (2015). Relationship of attention deficit hyperactivity disorder and postconcussion recovery in youth athletes. *Clinical Journal of Sport Medicine*, *25*(4), 355-360.	Not elite sport focused
42	Moran, R. N., Wallace, J., Murray, N. G., & Covassin, T. (2021). Effects of attention deficit hyperactivity disorder and learning disability on vestibular and ocular baseline concussion assessment in pediatric athletes. *Applied Neuropsychology: Child*, *10*(3), 276-282.	Not elite sport focused
43	Nelson, L. D., Guskiewicz, K. M., Marshall, S. W., Hammeke, T., Barr, W., Randolph, C., & McCrea, M. A. (2016). Multiple self-reported concussions are more prevalent in athletes with ADHD and learning disability. *Clinical journal of sport medicine: official journal of the Canadian Academy of Sport Medicine*, *26*(2), 120.	Not elite sport focused

44	Nguyen, T. (2017). Neurocognitive effects of gist reasoning training in student-athletes with concussions, ADHD, and learning disabilities (Unpublished doctoral dissertation). *Denton, University of North Texas*.	Dissertation
45	Nuwer, M. R., Nuwer, J. M., & Tsao, J. W. (2018). Student athlete concussions and postconcussion syndrome: ADHD as a risk factor. *Neurology: Clinical Practice*, *8*(5), 377-378.	Editorial/not elite sport focused
46	Rubinstein, J. B., McGrath, N., & Jackson, A. (2022). A-39 Baseline And Initial Post-Injury Presentation Of Athletes With Self-Reported Neurodevelopmental Disorders As Measured By ImPACT. *Archives of Clinical Neuropsychology*, *37*(5), 1080-1080.	Not elite sport focused
47	Salinas, C. M., Dean, P., LoGalbo, A., Dougherty, M., Field, M., & Webbe, F. M. (2016). Attention-deficit hyperactivity disorder status and baseline neurocognitive performance in high school athletes. *Applied Neuropsychology: Child*, *5*(4), 264-272.	Not elite sport focused
48	Bennett, R., Horne, K., Datoc, A., McDonald, B., & Lashley, L. (2020). A-05 Self-Reported Symptomatology in Athletes with Autism Diagnosis. *Archives of Clinical Neuropsychology*, *35*(5), 601-601.	Abstract
49	Bodine, W. A., & FAOASM, D. Autism Spectrum Disorder in the Pediatric Athlete.	Not peer-reviewed publication
50	Maietta, J. E., Renn, B. N., Goodwin, G. J., Maietta, L. N., Moore, S. A., Hopkins, N. A., ... & Allen, D. N. (2022). A systematic review and meta-analysis of factors influencing ImPACT concussion testing in high school and collegiate athletes with self-reported ADHD and/or LD. *Neuropsychology*.	Abstract
51	Poysophon, P., & Rao, A. L. (2018). Neurocognitive deficits associated with ADHD in athletes: a systematic review. *Sports Health*, *10*(4), 317-326.	Not elite sport focused
52	White, R. D., Harris, G. D., & Gibson, M. E. (2014). Attention deficit hyperactivity disorder and athletes. *Sports Health*, *6*(2), 149-156.	Not elite sport focused.
